# Evaluation of Subsampling Methods for Micro-Spectroscopy Analysis of Microplastics

**DOI:** 10.3390/toxics14100843

**Published:** 2026-09-22

**Authors:** Yifei Wang, Ujjwal Ghimire, Danny Reible, Balaji Rao

**Affiliations:** Department of Civil, Environmental, and Construction Engineering, Texas Tech University, Lubbock, TX 79409, USA

**Keywords:** microplastics, spectroscopy, subsampling, Monte Carlo Simulation, *Priori*

## Abstract

Full micro-spectroscopy analysis of micron-size microplastics (MPs) in environmental filter samples can be very time-consuming. Subsampling is necessary but leads to the introduction of uncertainty in the number, mass and type of MPs that have been collected on a filter. Commonly used random and stratified random subsampling makes minimal use of information about the sample and are compared here to subsampling approaches that make use of the particle number and size distribution of collected MPs obtained with fast pilot scans. These informed subsampling approaches bias the subsampling by proximity to mean count (PMC), proximity to mean size (PMS), proximity to areas with large counts (PPC), and proximity to areas with large size (PPS). These informed subsampling methods were benchmarked against Random and Stratified methods by Monte Carlo Simulations that concerned three metrics: (1) count estimation, (2) size profiling, and (3) identification profiling. For count estimation, the PMC method was marginally better than random methods. For size and identification profiling, the PMS outperformed all other methods. To reaffirm the practicality of the proposed methods, a demonstration was presented relying on PMC and PMS to execute the subsampling and estimate the overall count, size and identification of MPs. The results of this study make strong arguments for applying subsampling methods that make use of particle number and size distribution rather than random subsampling.

## 1. Introduction

Microplastics (MPs) pose a significant risk that affects ecosystems worldwide. There has been a lack of a consensus definition of MPs, but they can be broadly defined as any synthetic polymeric particle with sizes ranging from 1 μm to 5 mm that have been found in many ecosystems on the planet, and in food, beverages, and human and animal tissue [1,2,3,4]. Of particular interest here are micron-sized particles, which are found in large numbers in environmental matrices including air, water, soil, sediment and waste. Depending on sample type, a typical MP characterization workflow starts with sample processing including oxidative digestion, followed by collection on standard filters for analysis. While definitive human health studies are ongoing, exposure is linked to metabolic disorders, reproductive toxicity, and increased cardiovascular risks [3]. For these reasons, analysis of MPs in the environment warrants close attention. Thermo-analytics are capable of determining polymer types and total mass, while non-destructive spectroscopic techniques, particularly those relying on Fourier Transform Infrared (FTIR) and Raman spectroscopy, when combined with microscopy (as in micro-spectroscopy), are capable of providing information on not only type and counts, but also shape and size [5], all of which have implications for the toxicity mechanisms of MPs [6].

A major drawback for micro-spectroscopy analysis, however, is that it tends to be time-consuming and labor-intensive. A single filter can contain up to 10^4^ particles and consequently require an extensive amount of time to analyze even with automated spectroscopy methods. For instance, it took Pittroff et al. four to eight days to analyze 22.1% of a filter which contained 10,000–30,000 particles of sizes from 5 to 20 µm using semi-automated Raman microspectroscopy analysis [7]. Analysis time is also affected by the often accompanying large number of non-plastic particles associated with environmental samples, which may require further high-resolution imaging and spectra acquisition for individual particles [8]. One approach to mitigate long sampling time is to subsample a fraction of a filter and use that information to extrapolate to the entire filter.

Subsampling strategies reported in the literature involve measuring sub-section(s) of the entire filter areas, typically ranging from 0.5% to 50% [9,10,11], and extrapolating the subtotals to the whole filter. The strategies can be sorted into two categories based on whether or not the subsampling is guided by general information about the sample [12,13]. A *priori* subsampling refers to using available knowledge regarding some aspects of the sample in guiding the subsampling [14,15]. The strategies without a *priori* information are differentiated by the distribution of the subsampling areas, which can be modeled as (1) wedge, (2) cross, (3) spiral, (4) stratified, (5) grid, and (6) random [8,16,17,18,19]. Many studies adopted such strategies but often either lacked clarity in the rationale or failed to qualify the results by accounting for the error associated with such extrapolation [8,20,21]. Feng et al. offered a glimpse of associated potential extrapolation errors by demonstrating that grid-based subsampling of 4–8% area can lead to up to 17% count error [8], which does not seem disconcertingly large. However, the study relied on spiked MP samples excluding non-plastic particles, the presence of which could have in turn widened the error margin by impacting the deposition patterns of MPs. This was borne out by the study of Brandt et al. in which <10% of all filtered particles from environmental samples were MPs and, in many cases, subsampling at least 50% of the filter was needed to keep count error <20% [12]. These strategies’ lack of *a priori* knowledge regarding particle distribution and requires researchers to choose between prohibitive analysis time and significant statistical uncertainty. Without some knowledge of the sample, random sampling or stratified random sampling are generally more desirable than the systematic strategies (i.e., wedge, cross, spiral, and grid) in which locations are determined by a fixed geometric rule. This is mostly because the precision and accuracy of the systematic strategies depend on the homogeneity of the particle distribution on the filter, which is not verifiable in the absence of a *priori* [18,19,22]. Particles may accumulate at the edges or clump in the center, in which case the systematic subsampling can lead to consistent over- or under-estimation of the overall results. If random subsampling cannot be realized, it is recommended that small subsamples and numerous locations be preferred to large subsamples and few locations [18,22].

Subsampling strategies with *a priori* aim to establish a statistical foundation that enables researchers to minimize subsampling locations while maintaining accurate and precise extrapolation. Such a *priori* mainly takes two forms, i.e., epistemic and empirical. Epistemic *a priori* is universal knowledge that exists independently of the sample. One of the very few studies exemplifying the subsampling approach with epistemic a *priori* is the one by Cowger et al. [13]. The authors posited that the subsampled particle amount hinged on (1) tolerance of error margin and confidence interval when facing an unknown but likely vast population (i.e., >100,000 particles), (2) modeling subsampling particles as Bernoulli trials, and (3) that the resulting Binomial Distribution can be treated as a Normal Distribution for a population large enough based on the Central Limit Theorem [13,23,24]. For a pre-determined finite population, the authors argued for applying Finite Population Correction to adjust the required subsampling size [13,25]. By applying this epistemic *a priori* approach, for instance, to achieve a 10% error margin, with a standard 95% confidence interval, 96 subsampled particles would be sufficient for any large sample (i.e., containing >100,000 particles), or even fewer subsampled particles if given a finite known total population, e.g., 95 needed for a population of 10,000 (i.e., <1% overall) [13]. However, such an epistemic *a priori* approach is only as good as the applicability of the underlying assumptions. It first assumes uniformly distributed particles and cannot account for geometric bias such as clumping. It also fails to address the pitfall involving polymer-type minorities, which are likely to be entirely missed if only around 1% of the filter is scanned. With a scaling factor of 100, any tiny error in the subsampling will be magnified through extrapolation; as warned by Feng et al., analyzing less than 8% of the area risks high variation, i.e., low precision [8]. In a sense, epistemic a *priori* is less preliminary knowledge of the sample than a recipe to follow that is hardly foolproof. As for subsampling with empirical a *priori*, it employs exploratory practices to gather sample-specific data that inform more efficient subsampling. One such empirical practice is pre-scan [26], or pilot scan [19]. Lopez-Rosales et al. deposited samples on highly reflective Keveley slides to be pre-scanned, and swiftly recorded particle counts on each stripe of the slide using a laser direct infrared (LDIR) imaging system [26], thus obtaining knowledge of particle distribution. The study by Thaysen et al., on the other hand, recommended a full-scale pilot scan for a few filters to assess particle distribution to gain the particle distribution knowledge presumably applicable to all samples prepared the same way [19]. Nevertheless, neither study acted upon the empirical *a priori* so as to adjust subsampling size and location accordingly. In the Lopez-Rosales et al. study, the authors randomly selected and then characterized a subset of 40% of the overall surface and reported “excellent” overall count estimate, despite −14.1% to 21.2% recovery error, and “very satisfactory” overall recoveries, albeit conceding that the sparse distribution of minority types and sizes could severely skew the extrapolation. It is not unexpected that they reported high internal variations in extrapolated counts among the strips, a common phenomenon of random subsampling [8,11,12], which might translate to worse extrapolation results if a smaller subsampling size were to be taken for expediency. The authors stopped short of explicitly enacting *a priori*-based subsampling strategies, but recommended, based on the empirical *a priori*, discarding the strips on the edge that were sparsely populated to preempt underestimation and reduce workload. A more informed approach would be selectively subsampling fewer strips or grids that most closely match the overall density of the filter, which should be readily available after the LDIR scanning. The other study by Thaysen found high variations in particle counts among the squares that were subsampled following either a cross or random approach, which reflected low homogeneity, and adopted the stratified subsampling approach—randomly selecting from the inner circle and each ring one square. An arguably more sensible approach would be adjusting the subsampled area to the respective particle density within the circle and ring.

There is a strong demand for empirical a *priori* strategies that can considerably alleviate the workload while ensuring extrapolation fidelity to the true values. Such a strategy will streamline the MP analytical workflows that depend on micro-spectroscopy methods, which provide detailed chemical structural information—widely regarded as the gold standard for identifying and characterizing MPs [27]. In this study, we will employ the pilot scan via automated fluorescence imaging to develop a basis for informed subsampling. The pilot scan reflects the profiles of particle count and size but is not 100% accurate due to potential false positives from the co-staining of lipophilic compounds or substances other than MPs, such as natural lipids [28]. Subsampling methods using this information were then compared with random subsamples in a Monte Carlo-simulated particle size distribution on standard collection filters. Lastly, all the methods were tested out on a filter containing MP laden particles from an environmental sample. The goal was to benchmark and verify the a *priori*-based methods so as to apply them to expedite the micro-spectroscopy analysis of MPs confidently through a *priori*-guided subsampling strategies that are not spatial-blind (i.e., random) or spatial-fixated (e.g., cross), but attribute-informed, i.e., attuned to sample-specific characteristics regarding count and size.

## 2. Materials and Methods

The first sections detail the type of filter sample used to collect MPs and for which the subsampling seeks to represent the parent distribution of number, size, and identity of the MPs. This is also the method used to develop the actual sample used to demonstrate the proposed subsampling methods.

### 2.1. Standard Polymer Filter Preparation

Four types of standard polymers were used, which included low-density polyethylene (LDPE), poly(methyl methacrylate) (PMMA), and polystyrene (PS) obtained from a commercial vendor (Cospheric LLC, Goleta, CA, USA), and polyvinyl chloride (PVC) generated by cyromilling (courtesy of Dr. Jeffrey Farner, Department of Civil and Environmental Engineering, FAMU-FSU, Tallahassee, FL, USA), and featured two morphologies (i.e., sphere and fragment), a size range (1–32 μm) of high environmental relevance, availability, and instrument compatibility [29,30,31], and multiple colors. Details about the standard polymers are available in Table S1.

The standard polymers were used to make stock solutions for creating standard polymer-loaded filters. The preparation started with making a master stock by weighing roughly 2 mg of each polymer standard and adding it to 100 mL of 0.01–0.25% Tween 20, yielding an estimated concentration on the order of 10^5^ counts/mL. The working stock was created by diluting the master solution 1000 times in 100 mL of 0.01–0.25% Tween 20, achieving the concentration on the order of 10^2^ counts/mL.

Three aliquots of the same amount of working stock were pipetted onto three 13 mm-diameter glass fiber membrane filters of 0.3 μm nominal pore size (Sterlitech, Auburn, WA, USA) that were fitted to a conventional filtration system. The system consisted of a glass funnel, aluminum clamp, glass joint, receiving flask, and vacuum pump with the filter placed on the glass joint. The aliquots were dropped on the filters following specific procedures to create varying distribution characteristics, i.e., count variability and spatial correlation, to represent diverse filtration schemes. Specifically for the three standard polymer filters, #1: one drop of working stock on the central area of the filter; #2: several small drops across all four quadrants of the filter; #3: 10 mL 0.25% Tween 20 that contained one drop of working stock. Definitions of count variability and spatial correlation are given in Text S1. The spatial characteristics were determined based on true counts, not pilot scans. Each filter was then stained by directly applying 3–5 drops of low-concentration but fit-for-purpose Nile Red dye (62.5 µg/L in acetone) to the filter.

### 2.2. Environmental Sample Preparation

Biosolids were collected from a wastewater treatment plant by filling a pre-cleaned glass jar with a lid. The organic matter in the samples was then removed through hydrogen peroxide digestion—30% H_2_O_2_ at 65 °C for 72 h. Particles of light density in the digested samples were then separated by density through sequential density separation procedures, which, in brief, entail steps (1) that the digested biosolids went through centrifuge at 5000 g for 5 min, (2) that the resulting supernatant was vacuum filtered onto a 25 mm glass fiber filter (of 0.6 μm nominal pore size) for particles of light density, (3) that 5 mL ultrapure water (UPW) was then added to the remaining, and (4) that the centrifuge and collection processes were repeated to ensure most light-density particles were collected.

The filter of the low-density particles then went through the staining process by directly applying 3–5 drops of Nile Red dye (62.5 µg/L in acetone) to the filter.

### 2.3. Sample Scanning and Processing

Each filter was secured on a glass slide and loaded into the sampler chamber of an optical photothermal IR spectroscopy (O-PTIR) (mIRage-LS™ Infrared microscope, Photothermal Spectroscopy Corp., Santa Barbara, CA, USA). The system was operated under ambient laboratory conditions and was automatically background-calibrated to reduce interference when the humidity changed by more than 2%. For each field of view (FOV) (1024 µm × 1024 µm) under a 10× objective, the image was first autofocused and, if needed, further manually focused. Then particle counts and sizes were automatically tallied based on thresholds of fluorescence intensity and exported in spreadsheets through the built-in FeaturefindIR^®^ (PTIR Studio, version 5.0.1.9705) application. The objective then moved on to the next FOV, and the same procedures were conducted. The process repeated itself until the whole filter was scanned, yielding about 120 individual spreadsheets for the 13 mm standard polymer filters, and 500 for the 25 mm environmental sample filter, containing particle global coordinates and size within each FOV, which concluded the pilot scan that generated the sample-specific a *priori*. Each individual pilot scan took approximately one and a half hours. The true particle counts were determined by auditing each FOV for unidentified and misidentified particles caused by inaccuracy of software algorithm and auto-fluorescence of filter materials [32]. The polymer type was determined through procedures, the first of which was the IR spectrum acquisition. Using a 40× Cassegrain objective, a pulsed Quantum Cascade Laser was focused on the particle to induce the photothermal expansion that was simultaneously detected by the visible probe to generate the IR spectrum, which was then exported in the wavelength-intensity format to a spreadsheet for processing. The QCL power was kept below 10%. Cross-reference of spectra retrieved from the instrument was performed with the open-source database Open Specy version 1.5.3 [33].

### 2.4. Subsampling Methods

Six methods were selected, which included Random to represent the spatial-blind and Stratified to represent the spatial-fixated. The remaining four are attribute-informed empirical a *priori*-based methods: (1) proximity to mean count (PMC), (2) probability proportional to count (PPC), (3) proximity to mean size (PMS), and (4) probability proportional to size (PPS). The technical approaches of each method in determining subsampling locations and estimating the characteristics of the entire filter are summarized in Table 1; an exemplary demonstration is illustrated in Figure 1 (produced by Python_script_0, Supporting Information). The explicit mathematical formulas employed by each method are available in Table S2. The a *priori* was derived from the pilot scan, as described in the previous section, which contained information on particle counts and sizes. As suggested in the table, Random and Stratified did not involve an a *priori*, in either subsampling or estimation. PMC and PMS depended on a *priori* information to subsample but not to estimate, while PPC and PPS utilized a *priori* information throughout.

### 2.5. Monte Carlo Simulations

Monte Carlo (MC) simulation of 1000 iterations was executed in a Python 3.14 environment to compare the six methods based on three aspects of the sample, namely total count, size profile, and polymer type profile, with specific metrics. For total count, mean relative error (MRE) served as the sole metric; for size profile, MRE of span was used to compare accuracy, relative standard error (RSD) of $D_{50}$ for precision, and Spearman’s coefficient size for rank fidelity; for polymer type profile, mean absolute error (MAE) of identification proportions was used to compare accuracy, RSD of the most common polymer identification for precision, and Spearman’s coefficient identification for rank fidelity. The explicit mathematical formulas are available in Texts S2 and S3. A generic version of the Python script in each step is available in Supporting Information. All the related spreadsheets are also available in Supporting Information. Random and Stratified participated in all tournaments. PMC and PPC, which were conceived for the count and guided by the count, did not take part in the MC tournament of size profiling. By the same token, PMS and PPS were absent from the tournaments of count estimation. Nevertheless, all six methods attended the tournament of polymer type profiling. The subsampling sizes (*n*) involved in the MC tournament were set in ranges that covered the subsampling size that accounts for roughly 10% of total locations, i.e., n ≈ 15 and 50 for the 13 mm standard polymer filter (with 126 total locations) and for the 25 mm biosolid sample filter (with 468 total locations), respectively. A total of 10% represented a tentative, exemplary subsampling size in this study to drastically save time while maintaining veracity.

## 3. Results and Discussion

### 3.1. Standard Polymer Filter

The six subsampling methods first competed among each other in the MC tournaments with the three standard polymer 13 mm filters. The collated and processed data of the three pairs of pilot scan and ground truth (GT) are available as spreadsheets in the Supporting Information. The mean local false positive ratio (FPR), its definition, its relative standard deviation (RSD), and the characteristics of particle distribution for the three filters are available in Table S3.

### 3.2. Count Estimation

Random, Stratified, PMC and PPC participated in the MC tournament on count estimation (carried out by Python scripts 1–4 and 5, Supporting Information), the results of which were based on RSE. As shown in Figure 2, the two a *priori*-based methods performed drastically differently. PMC marginally led over the rest with around the subsampling size target (i.e., 10 < n < 15) on two of the filters (Figure 2a,c) but struggled on the other (Figure 2b). The other a *priori*-based method, PPC, on the other hand, fared remarkably worse, ranking last in terms of MRE of counts and variance on all three filters, especially on two of them (Figure 2a,c), despite the fact that it was designed to leverage count-related a *priori* information for better count estimation.

PPC relied on the pilot scan-based probability not only to determine subsampling locations but also in the Horvitz–Thompson (HT) estimator to calculate the total count [34] (Table S2). It is based on the pilot scan to assign selection weights, which were then inversely used in the HT estimator for overall counts (Table S2). The design was to make the preferably selected locations exert a smaller effect on total counts; therefore, to cancel out the selection bias and produce robust and reliable overall estimation [35]. However, when FPRs were not consistent among the locations (i.e., high RSD), with some locations assigned selection weights either greater (i.e., higher PFR) or smaller (i.e., lower FPR) than supposed to be (i.e., mean FPR), the weight-based count calculation led to either overestimation (due to lower FPR) or underestimation (due to higher FPR). As attested by the largest MRE in Figure 2, the estimation bias in both directions did not cancel out and stabilize the estimation. This mainly resulted from the confluence of highly inconsistent FPRs (i.e., high RSD) and finite subsampling size. Wildly fluctuating FPRs inevitably led to a subsample consisting of overestimated locations and underestimated ones, with the overall estimation ending up being either greatly overestimated or the other way around, because of the limited subsampling size—the point being that bias in both directions should balance out if all locations were sampled. Although all the subsampling methods were dealing with the same pilot scan data in which FPRs were intrinsic, only PPC was especially sensitive to while others were inherently less susceptible to the whims of FPR.

Random and Stratified, true to the non-*priori* methods, subsampled solely based on locations (Table 1), disregarding pilot scans, thus immune to FPR. They also used a fixed expansion factor for estimation (Table S2). PMC, which preferably selected locations with counts close to the global means, did subsample under the influence of FPR. But the effect was moderate primarily because counts close to the global means generally were not outliers even with varying FPRs. In other words, PMC focused on the more representative central sample set of the pilot scan distribution that did not lead to erratic estimation swings. In addition, PMC estimated the total counts following the same algorithm as random, shielding itself from further FPR influence during estimation after subsampling. In comparison, highly inconsistent FPRs were a double whammy to PPC, misguiding both subsampling and estimation, which resulted in outcomes of sizable error and variance.

Comparing among the three standard polymer filters in Figure 2, it is safe to say that particle distribution had more prominent effects on some methods than on others. Judging from CV and Moran’s I, particles were distributed with the least count variability on filter #3, the most on filter #2, and moderately on filter #1, while the spatial correlation, i.e., patchiness, was markedly stronger on filter #2 than the other two (Table S3). PPC was expectedly the worst performer on filter #1, which displayed the highest RSD of FPR, to which PPC remained hypersensitive and beholden because the inconsistency of FPRs was baked into its processes and inevitably led to highly skewed estimations, as previously discussed. Its more underwhelming performance with filter #3 was surprising because filter #3 exhibited the lowest RSD of FPR. This may be partly due to its lowest count variability.

The effect of count variability can be better explained together with the notably better performance of PPC on filter #2 (Figure 2b) despite similar FPRs and RSDs of FPR with filter #1 (Table S3). It also coincided with relatively subpar performance by PMC on filter #2, in comparison with itself on the other filters. The main reason responsible for this had to do with the statistical peculiarity created by the specific particle distribution and high-RSD FPRs on filter #2. The particles on filter #2 were most polarized, attested by the highest CV by far, meaning most locations were either highly or sparsely particle-populated. This led to a mostly hollowed-out portion of medium-count locations from which PMC mainly subsampled because their counts were close to the global means. The extremely high CV translated to an exceedingly limited pool size of such PMC-qualified locations that, in the meantime, inevitable become less truly representative of the global means. This problem of diminished quantity and quality was compounded by the high-RSD FPR among the medium section. This is because (1) the FPRs among the vast majority of low-count locations were capped below 1 due to the fact that the pilot scan systematically overcounted, and (2) the FPRs among the high-count locations were highly self-stabilizing with large pilot scan counts (i.e., large denominators in FPR calculation [36]). The corollary is that the highly fluctuating FPRs in the medium-count section were most responsible for the high RSD. Therefore, to summarize, high CV compromised the representative power of PMC that was further aggravated by the widely oscillating FPRs.

Conversely, the extremely high CV of filter #2 was a boon for PPC, as PPC performed evidently better than on the other two filters (Figure 1). The main reason is that PPC calculated the selection probabilities based on the local count of the location, which means that, when the extremely high CV led to extremely high counts, those high-count locations with overwhelmingly high selection probabilities hoovered up most subsampling slots. With the relatively stable FPRs, as discussed above, the count estimation by PPC was comparable with others in terms of MRE, but the mathematical and algorithmic vulnerability of its functions (argued in the preceding paragraph) still led to unsettlingly high variance (Figure 2b). Conversely, when CV was low, as in the case of filter #3, particles were more numerically evenly distributed, as were the PPC selection probabilities. As a result, PPC subsampled more locations of lower counts that also had more volatile FPRs than the high-count locations.

Comparing filter #2 with the rest, Random was seen to have improved the most. Although the better estimation was accompanied by both lower CV and Moran’s I (Table S3), it was the lower CV that was solely responsible. This is because Random fundamentally was a spatial-blind approach that was technically immune to any effect brought by shuffling the grids, which means that locations of high/low counts were more removed from their (high/low-count) cohort in the case of lower Moran’s I, because each location always had an equal chance of being subsampled by Random. The performance gain seen in Random was, therefore, mainly thanks to the lower count variability. When count variance (i.e., variability) decreased, the probability increased that a Random subsample included fewer outliers but more representative ones reflective of the global means. Similarly, lower count variability also benefited Stratified in that stratum-specific subsamples were more likely to contain more representative locations. However, the drop in Moran’s I, i.e., less clustering, acted as a structural penalty for Stratified. The core mechanism of Stratified makes it thrive on low intra-stratum variance [37]. Such a feature enables Stratified to stand out especially when facing high inter-stratum variance, such as the case of high Moran’s I, in which a major portion of any cluster of particles was likely to reside within one stratum. As particles got less clustered and more dispersed, the localized clusters fragmented into scattered distributions, causing the inter- and intra-stratum variances to converge, thus dampening its edge and making its performance less distinguishable from Random (Figure 2a,c).

Apart from the special case of extremely high CV and high RSD of FPRs (as with filter #2), PMC was arguably the frontrunner given the below 20% MRE with filters #1 and #3. PMC considered only the count attribute during subsampling, making it immune to changes in spatial correlation (i.e., Moran’s I) but receptive to the benefit brought by lower count variability, which meant fewer extremes in the subsample and better representation as CV dropped. Everything considered, PMC was chosen as the subsampling method for count estimation.

### 3.3. Size Profiling

Random, Stratified, PMS and PPS participated in the MC tournament on size profiling (carried out by Python scripts 1–4 and 6, Supporting Information), the results of which were based on MRE of span for accuracy, RSD of $D_{50}$ for precision, and Spearman’s coefficient (ρ) for size rank fidelity, all three of which are mathematically defined in Text S2. As shown in Figure 3, PMS swiped the series—outperforming the rest in accuracy, precision, and size rank fidelity across the subsample sizes. At *n* = 15, around the subsampling size target of 10% total locations, it achieved less than 20% MRE of span, which was the proxy for the width and height of the size distribution curve [38], indicating high accuracy in estimating the shape of the curve; below 10% RSD of $D_{50}$ proved low variance; a greater than 0.9 Spearman’s coefficient confirmed the reliability of its size profiling.

PMS was designed to preferably subsample from an eligible subgroup of locations, the size distribution of each one of which was at least 75% akin to the global size distribution in the pilot scan in terms of size ranking (i.e., Spearman’s coefficient) (Table S2). To obtain the global size profile, the size profiles (i.e., histograms) of the subsampled locations were then aggregated element-by-element (i.e., size bin-by-size bin), followed by the simple expansion method based on the subsample size (Table S2). PPS arguably ranked second best to PMS. It also fared notably better in this MC tournament than its counterpart, namely PPC, did in the other MC tournament on count, even though the highly volatile FPRs were universal—the size profiles estimated by PPS were highly accurate, precise, and truthful to rank as shown in Figure 3, in contrast to PPC (Figure 2). There are two primary reasons behind the divergence. The first is that, although PPS subsampled according to the pre-determined probability as PPC (based on different attributes), PPS was adopted for estimating the size profile Hájek projection framework [39] (Table S2), which was largely immune to massive, varying FPRs because the total particle size sum of the pilot scan ($\sum s_{i}$, Row PPS, Table S2), which the FPRs were baked into, canceled out when substituting out the probabilities ($\pi_{i}$, Row PPS, Table S2), whereas the total particle count of the pilot scan ($\sum w_{i}$, Row PPC, Table S2) stayed with the HT estimator for PPC after substitution and subjected PPC to the heavy influence of FPRs. The PPS design remained active during profiling by keeping the local scan ($s_{i}$, Row PPS, Table S2) after the substitution. The second reason is that PPS evaluated intensive metrics, which remained relatively stable when datasets were combined, whereas PPC evaluated an extensive metric (i.e., count), which was scaled up, while also multiplying errors (i.e., FPRs), by the HT estimator. The intensive metrics, namely span, $D_{50}$, and size rank, were structural parameters of the cumulative distribution function (CDF) for size distribution, which was a self-normalized, bounded curve. The metrics described the shape and width of the CDF curve, in which a localized error (e.g., erroneous relative abundance of particles in a certain size bin) could only warp a portion of the curve with limited effect on the curve as a whole.

PMS outperformed PPS mainly because the subsampling vehicle of PMS was aligned with the MC tournament metrics—PMS subsampled relying on the proxy of size rank (Table S2), which was not only itself a metric in the MC tournament but also closely related to span and $D_{50}$, the other two structural parameters. PPS, on the other hand, subsampled based on the extensive metric of total particle sizes, which was not well calibrated to the three intensive metrics. PMS also steered clear of the edge cases that PPS was blindly susceptible to, such as the locations of a few large-size particles and numerous small-size particles, both of which were not representative of the global size profile. Nevertheless, it is notable that both a *priori*-based approaches, i.e., PPS and PMS, outperformed the non-a priori, i.e., Random and Stratified, on all three metrics with a sizeable margin except for accuracy on filter #2 (Figure 3), compared with the mixed results from PPC and PMC (Figure 1). The reason why PPS still convincingly outran Random and Stratified is that its subsampling approach, based on high total particle sizes, bypassed most empty and sparsely populated locations and therefore greatly boosted the chances of high-count subsampled locations. High count was important because the size profiling, in terms of the three metrics, was closely linked to the shape of the CDF size distribution curve, which benefited from the law of large numbers (LLN) [40], and high count from PPS translated to a relatively smoother and less jagged CDF curve than the Random and Stratified, both of which indiscriminately subsampled and inevitably included the zero- and low-count locations that degraded the CDF curve. Furthermore, PPS was also shielded from escalating effects of volatile FPRs by the Hájek estimator, as previously argued.

Judging among the three filters, the characteristics of particle distributions and RSD of FPRs had less effect on size profiling than previously on count estimation. Stratified performed relatively moderately better on filter #2 (of highest Moran’s I) than on the other two filters, with higher accuracy ranking and greater precision margin over Random (Figure 3b). The modest performance bump could be attributed to the same reason why Stratified estimated counts more truthfully with filter #2 (Figure 2b), which is that the combination of low intra-stratum variance and high inter-stratum variance resulting from high-degree clustering (i.e., high Moran’s I) worked in favor of Stratified, as previously argued. Other than that, despite varying count variability (in terms of CV) and RSD of FPRs (Table S3), the performance ranks did not see a significant shift—PMS consistently ranked first overall and Radom last. In fact, the rankings of size rank fidelity remained unchanged throughout. This is because (1) count variability, as titularly indicated, describes how numerically evenly distributed the counts are, not the particle sizes per se, and (2) the FPR was arguably size-neutral given that the false positives, in the case of standard polymer filters, originated from the systematic error due to the failure of software to accurately screen for the stained MP particles against the occasionally bright background lit up by the co-stained organic binders composed in the glass fiber filters [28,41], which was not size-related. The most notable outcome of this MC tournament on size profiling is the consistently superior performance of PMS regardless of CV, Moran’s I, and RSD of FPR. PMS was therefore the undisputedly preferred subsampling method for size profiling.

### 3.4. Identification Profiling

Since no a *priori* information could be gathered regarding the polymer identification in the pilot scan, all six subsampling methods participated in the MC tournament on identification profiling (carried out by Python scripts 1–4 and 7, Supporting Information), the results of which were based on mean absolute error (MAE) of identification proportions for accuracy, RSD of the most common polymer identification for precision, and Spearman’s coefficient (ρ) for identification rank fidelity, all three of which are explicitly defined in Text S3. The MC tournament opted for MAE, instead of MRE as with the one on size profiling for span, because some polymer types were present only at a trace level, namely an extremely small denominator in the equation for the MRE of identification proportions, in which case a small absolute error (in the numerator) would register a staggering error and cause the error curves to spike due to minor miscalculation regarding trivial polymers.

As shown in Figure 4, PMS produced the most accurate identification profiling with filters #1 and #2, although with filter #3, its performance was mixed—excellent in profiling the most common polymer type (i.e., RSD of Key ID) but underwhelming in terms of the other metrics. The strong showing of PMS was not expected because more representative size profiling did not necessarily benefit identification profiling since no standard polymers used in this study featured a full size range, as shown in Table S1. Nonetheless, the dominance of PMS in identification profiling was due to one of the main reasons that made it successful in the size profiling, which is the LLN. As discussed in the previous section, the algorithm of PMS subsampling automatically sieved out most low-count locations because there needed to be enough particles to occupy the size bins to yield a qualifying continuous size distribution curve, the shape of which tended to converge with the true global curve as particle count increased according to LLN [40]. Although the identification distribution is a discrete categorical frequency distribution, not a continuous histogram, LLN applied as powerfully with multinomial convergence, which dictated that the proportions of individual polymer types in the subsample converge to those true proportions as the particle count grew [42]. The other three a *priori*-based methods fell short of taking advantage of LLN as effectively. PPC assigned high selection probabilities to high-count locations and spent most subsampling budget on them, poised to benefit from LLN; however, its Hájek estimator was by design probability-weighted and mathematically flattened volumes to average probabilities (Table S2), e.g., equalizing the weights of the 30-count location that had 10 particles of a certain polymer type with the 3-count location that had 1 polymer of that type, which forfeited the LLN advantage. In the same vein, PPS, which also employed the Hájek estimator, could not claim the LLN benefit. Additionally, since PPS subsampled under the guidance of total particle sizes, it remained vulnerable to the edge cases of low-count large-size locations, as in the previous MC tournament in size profiling. The low-count locations were inherently unlikely to embody the global polymer categorical frequency distribution, thus compromising PPS’s performance. As for PMC, it was proposed that sample size was the main reason for its inconsistent performance, i.e., decent results on filters #2 and #3 but objectively dismal results on filter #1. PMC only subsampled locations with counts close to the global means, which restricted access to the high-count locations that were critical for composing the polymer categorical frequency distribution. As a result, PMC risked more likely missing out on the high-count locations than Random and Stratified, depending on the specific particle numerical distribution. As attested by filter #1, PMC was demonstrably unreliable for polymer-type profiling.

Varying degrees of count variability, spatial correlation, and volatility of FPR did not seem to influence the overall performance of methods in one way or the other. The vapid effect was comparable to that seen in the MC tournament of size profiling, which was largely immune to the swinging of CV, Moran’s I, and RSD of FPR because, as discussed above, the characteristics of particle distribution were particle size-irrelevant, and FPR was particle size-neutral. An even stronger argument can be made for the minimal effect of such particle features on the performance of the subsampling methods in identification profiling, because none of the a *priori*-based methods featured polymer identification in their algorithms, as no such related a *priori* was available during the pilot scans. Filters #1 and #2 represent two drastically different degrees of homogeneity related to particles on the filter—filter #1 with a more even particle distribution (i.e., lower CV and Moran’s I) and filter #2 with a markedly less spread-out particle distribution. Therefore, the consistently superior performance put up by PMS on both filters made it the preferred subsampling method for identification profiling at the subsampling size of 10% total area.

### 3.5. Environmental Sample

The biosolid sample filter was used to (1) test and verify the preferred choices of subsampling methods, and (2) demonstrate how to estimate the overall count, size profile, and polymer type composition profile based on the subsamples. The biosolid sample filter was different from the standard polymer filters in filter size (~500 vs. ~150 total locations), an exceedingly high false positive ratio (98%) and RSD of FPR (>200%), along with extreme characteristics of particle distribution in terms of count variability (CV > 200%) and clustering (Moran’s I > 0.58) (Table S3). First, the same three series of MC tournaments were held for the biosolid sample filter (carried out by Python scripts 1–7, Supporting Information), and the results are shown in Figure 5.

The only result that did not agree with the standard polymer filters’ preferred choices was that of the MC tournament of count estimation (Figure 5a). PPC performed exceptionally well, achieving the smallest MRE of around 17% at a subsampling size of 10% total area, i.e., *n* = 50. The result was not entirely surprising when compared to the count estimation result on filter #2, which possessed the highest CV among the three standard polymer filters (Figure 2b). As the explanation for the relatively better performance of PPC on filter #2 was that extremely high CV created a group of locations with massive particle counts and forced PPC to spend the vast majority of the sampling budget on them, a similar but even more high-count-focused subsampling by PPC took place with the biosolid sample filter of higher CV. The remarkably high mean local FPR (98%, Table S3) did not weigh down PPC largely thanks to its being significantly uniform (5.08% RSD, Table 1). When FPRs among high-count locations deviated only slightly away from the mean FPR, the local selection probabilities assigned by PPC according to pilot scan became nearly proportional to the local particle count share in ground truth. This made the estimation by PPC not only more truthful to the ground truth, but also with less variance. Nevertheless, the choice of the standard polymer filters, i.e., PMC, did not lag far behind, achieving <20% MRE with only around 3% separation from PPC at *n* = 50. As for size and identification profiling, the results mirrored those from the standard polymer filters—specifically, PMS was the best performer for both. It is remarkable that PMS output had comparable or even higher accuracy, precision, and rank fidelity with the biosolid sample filter than with the standard polymers, in spite of high heterogeneous particle distribution, attested by the highest CV and Moran’s I (Table S3), and high FPR, as shown in Table 2. Therefore, the preferred subsampling methods for size and identification profiling determined through the three standard polymer filters were verified for PMS. Although PMC conceded to PPC in count estimation with the biosolid filter for the best a *priori*-based method (Figure 5a), it remained the preferential choice given the major drawbacks of PPC, including that (1) the reliable performance of PPC hinged on highly specific particle distribution features (i.e., extremely high count variability), and (2) the algorithmic design of PPC, although mathematically valid in theory and occasionally highly accurate in practice, was inherently sensitive to fluctuating FPRs as discussed and seen in the MC tournament of count (Figure 2).

To demonstrate how to deploy the chosen a *priori*-based subsampling methods to analyze a sample in practice, PMC and PMS were executed step-by-step to estimate the overall count, size profile, and polymer-type composition of the biosolid sample filter. The schematic of the demonstrative workflow is shown in Figure 5.

The first and foremost procedure was to estimate the overall count (within red confines, Figure 6), which would be needed for size and identification profiling. A total of 50 subsampling locations were selected by PMC based on the local count and global average count of the pilot scan (carried out by Python scripts 1–4 and 8, Supporting Information). The corresponding counts of the subsampled locations, which were identified by coordinates, were retrieved from the ground truth and estimated (following the formula in Table S2). The error margin was obtained from the MC tournament of counts (Figure 2). The count estimation by PMC proved demonstrably truthful to the real particle count (393 vs. 396).

Size and identification profiling were both taken on by PMS. Another set of 50 subsampling locations, as shown in Figure 6, was selected by PMS based on the local size rank and global size rank of the pilot scan (carried out by Python scripts 1–4 and 9, Supporting Information). For size profiling (within blue confines, Figure 6), the corresponding size histograms of the subsampled locations were retrieved from the ground truth and aggregated size bin-by-size bin, and the proportions of the size bins were then calculated. Lastly, the particle count previously estimated by PMC was applied, and the particle counts of the size bins were calculated. For identification profiling (within green confines, Figure 6), the corresponding proportions of polymer types were retrieved, aggregated, calculated from the same set of subsampling locations, and converted to particle counts according to the PMC-estimated total count. The PMS accurately projected the size rank for particles smaller than 50 µm, which accounted for around 98% of the true total count, and the estimated proportion for each of the five size bins came with <25% relative error. The identification profiling was expectedly more challenging than PMS, as PMS was not tailored for it by design; nonetheless, its projected polymer-type rank was accurate for the three most numerous types of polymers. The estimated counts for the top two polymer types were within 10% relative error. The total estimated counts for the top six polymer types, which accounted for around 90% of the true total count, were 355, exceedingly close to the true count of 356. The results confirmed the practicality and robustness of the proposed approach under realistic analytical conditions. The entire process, from pilot scan to estimation and profiling, was estimated to last 2.5 h. In comparison, if the whole filter were fully scanned and characterized in lieu of subsampling, each FOV would take around 0.5 h, which means that the total processing time for the 500 FOVs would amount to around 250 h, namely 100× more time-consuming than the demonstrated subsampling approach.

## 4. Conclusions

This study set out to design, test, and verify a *priori*-based subsampling methods that presumptively would produce more truthful estimates than their non-a priori-based counterparts, given the a *priori* derived from a simple pilot scan of any guide. Through the MC tournaments on three filters prepared by standard polymers of known quantity and features, two out of four a *priori*-based subsampling methods emerged as clear top performers—PMC for count estimation and PMS for both size and identification profiling. The finding is serendipitous, as the single master subsampling method was the tentative target at the outset, but verily unprecedented—different subsampling methods apply to different aspects of the MP sample. Their being the preferred subsampling methods was subsequently mostly borne out by the MC tournaments on a biosolid sample filter. The subsampling and estimation processes by the two chosen methods were also demonstrably practical. The demonstration with the biosolid environmental sample attested to the accurate and precise estimates based on 10% total sampling locations by the two a *priori*-based methods, propitious for the prospect of drastically reducing the workload and shortening the turnaround time for micro-spectroscopy analysis of MP with confidence.

It is herein recommended that a *priori*-based subsampling methods be adopted for micro-spectroscopy analysis of microplastics, specifically applying PMC for estimating the overall particle count and PMS for profiling particle sizes and identifications. Although this study took on 10% subsampling size, researchers should set the subsampling size according to the study’s priorities, standards, and resources, among other considerations. This study clearly laid out a practical roadmap consisting of the following steps: (1) rapid, simple pilot scan, (2) PMC for count estimation, and (3) PMS for size and identification profiling. This study employed FeaturefindIR^®^ application by the O-PTIR maker of Photothermal Spectroscopy Corp., for pilot scans to rapidly capture the locations and size of particle candidates. Such pilot scans can also be achieved on many other contemporary analytical platforms such as Agilent Clarity Software by Agilent and MP-ID Microplastics Identifier by Bruker^©^. Therefore, the verified subsampling methods in this study, composed of pilot scans followed by a *priori*-based procedures, are arguably highly transferable and likely to help alleviate one of the most challenging bottlenecks in MP analysis, i.e., time constraint, to expedite MP study and advance its understanding.

## Figures and Tables

**Figure 1:**
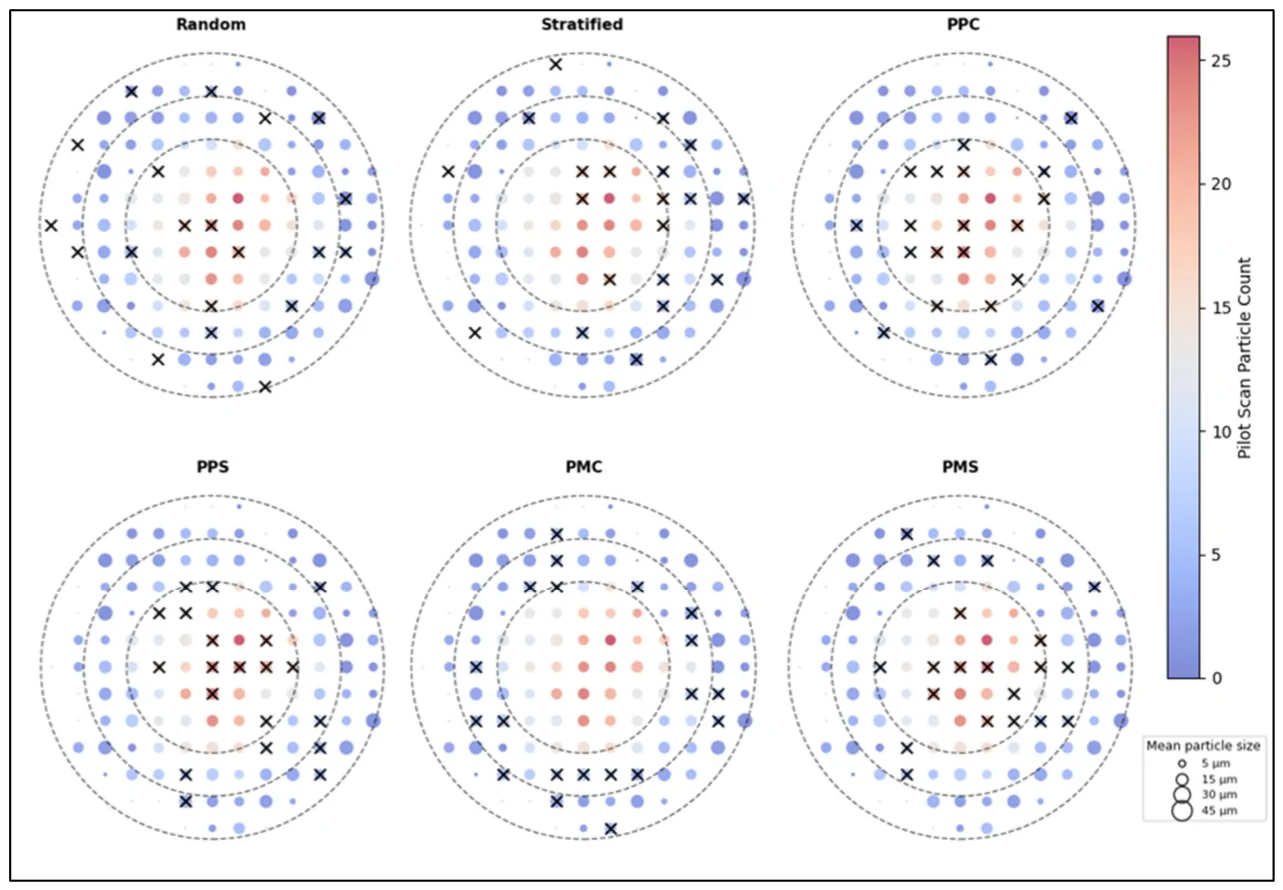
Exemplary demonstration of each method on subsample location (marked by cross) selection (*n* = 20) using an artificially generated filter with more particles (indicated by a redder hue) near the center. The mean particle size is indicated by the location size. The black dashes delineate boundaries of 0.5, 0.75, and 1.0 radii of the filter.

**Figure 2:**
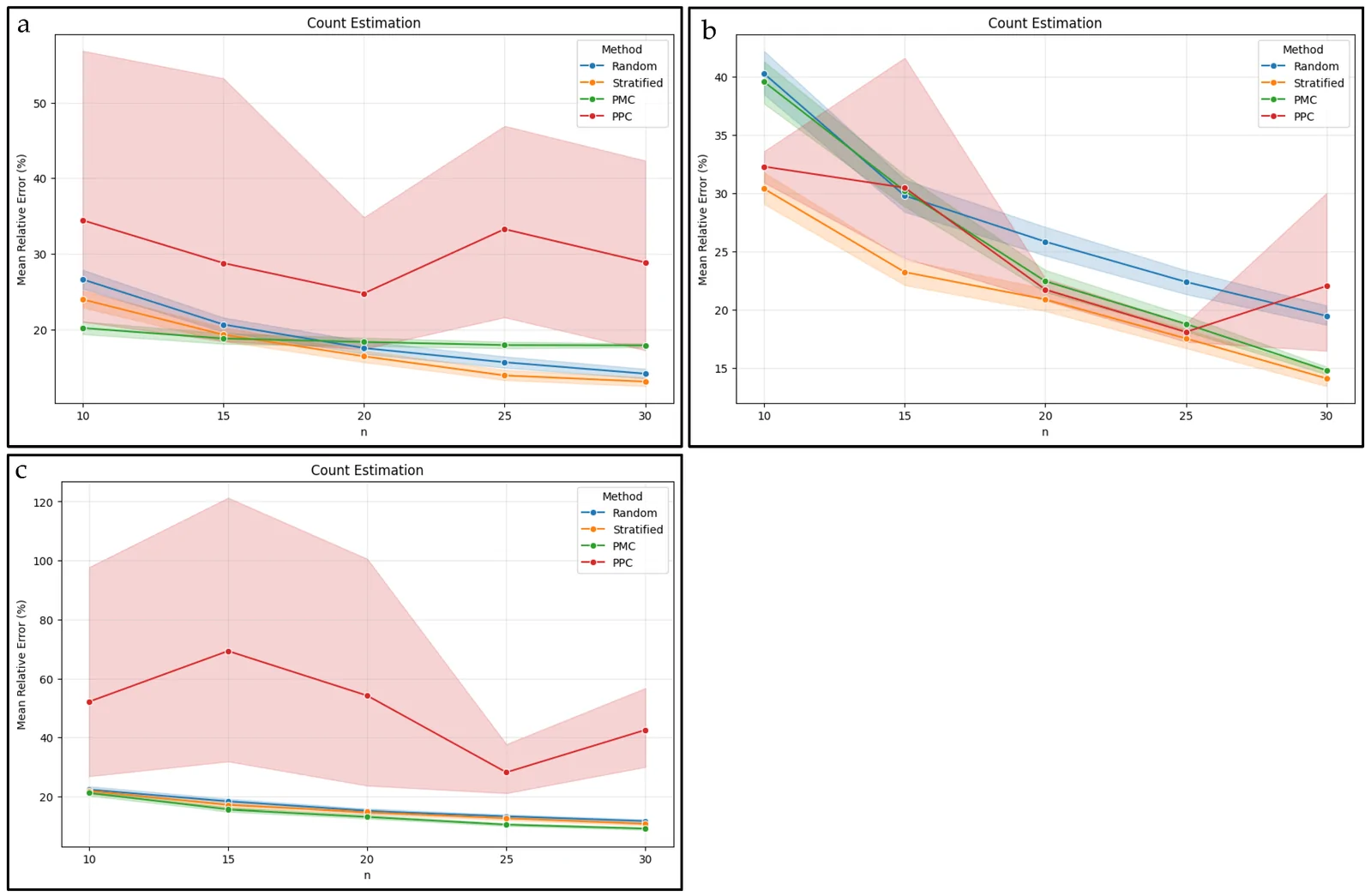
Results of MC tournaments on count estimation from three standard MP filters—#1 (**a**), #2 (**b**), and #3 (**c**). Subsampling of *n* = 30 represents 25% of the total filter sampling area. The shaded bands surrounding each method’s line represent the 95% Confidence Interval of the mean.

**Figure 3:**
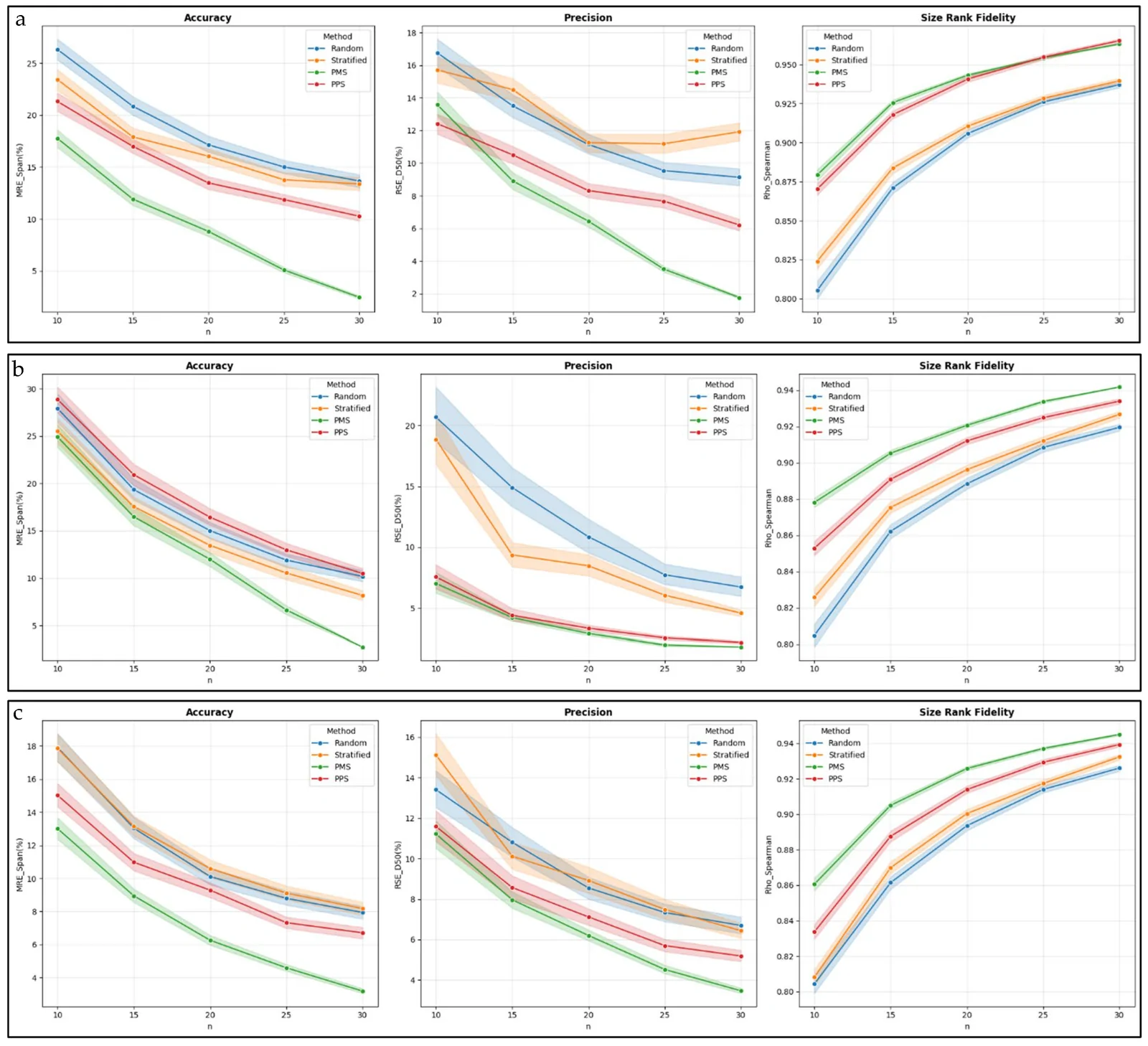
Results of MC tournaments on size profiling from three standard MP filters—#1 (**a**), #2 (**b**), and #3 (**c**). The shaded bands surrounding each method’s line represent the 95% Confidence Interval of the mean.

**Figure 4:**
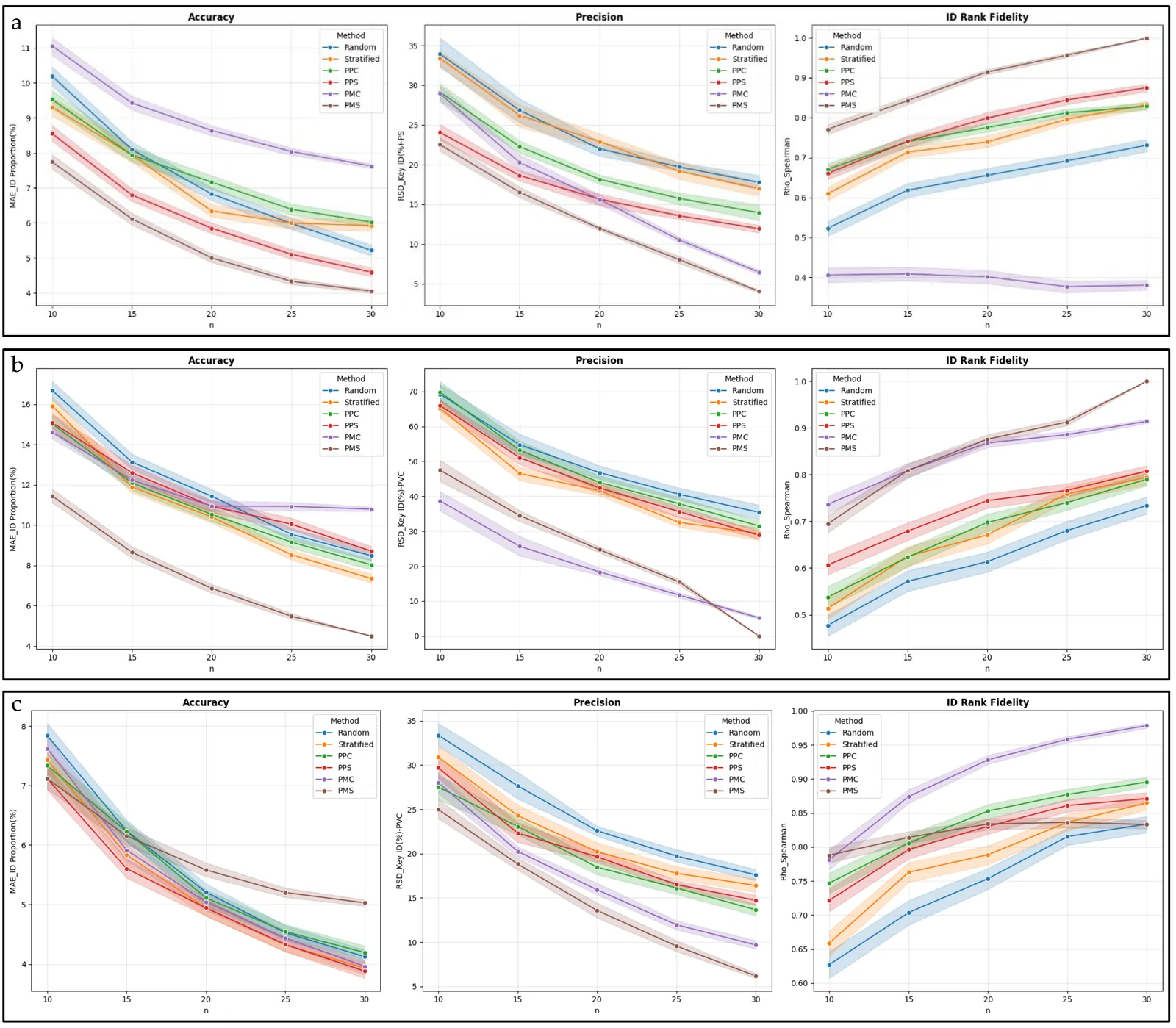
Results of MC tournaments on identification profiling from three standard MP filters—#1 (**a**), #2 (**b**), and #3 (**c**). The shaded bands surrounding each method’s line represent the 95% Confidence Interval of the mean.

**Figure 5:**
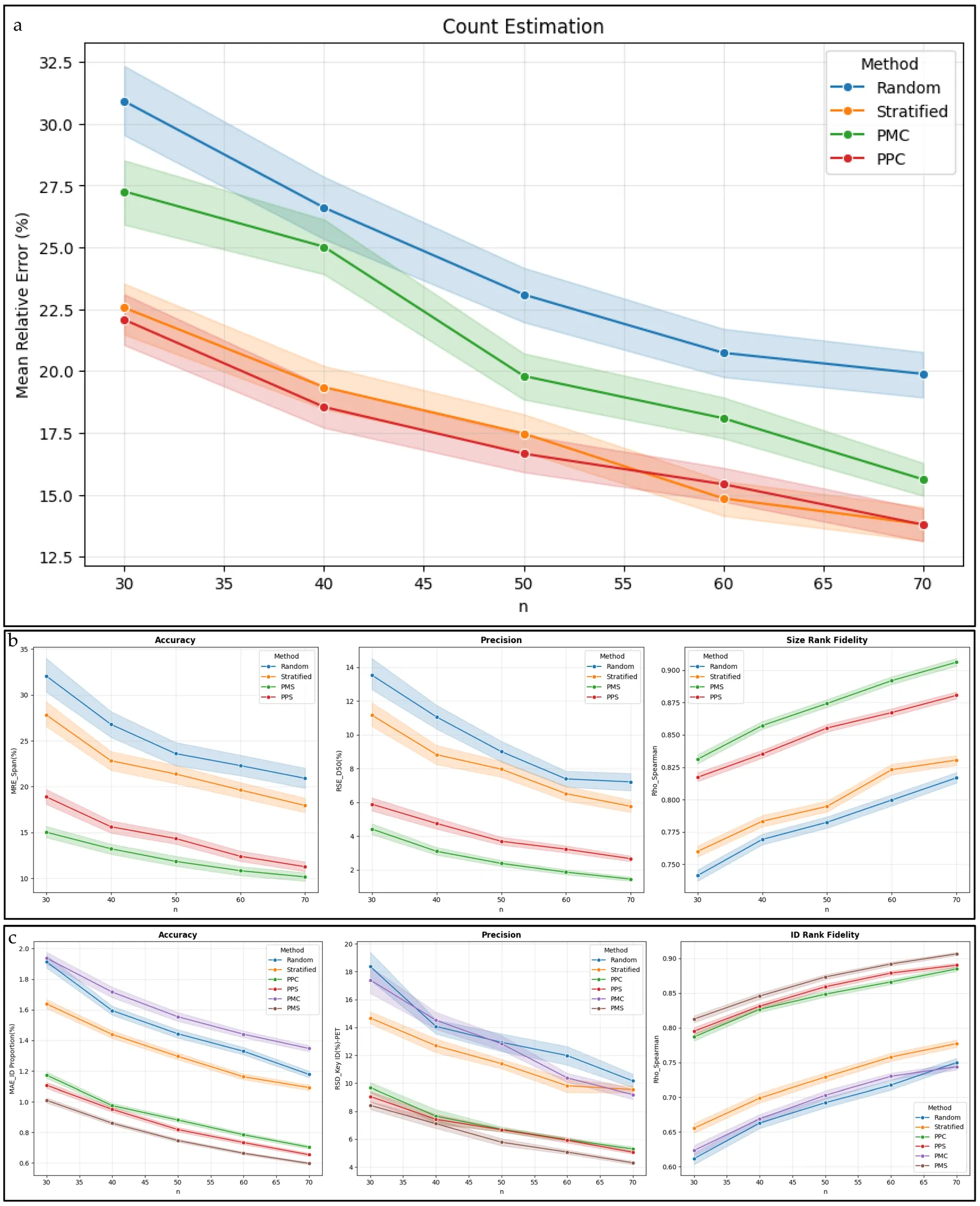
Results of MC tournaments from the biosolid sample filter on count estimation (**a**), size profiling (**b**), and identification profiling (**c**). The shaded bands surrounding each method’s line represent the 95% Confidence Interval of the mean.

**Figure 6:**
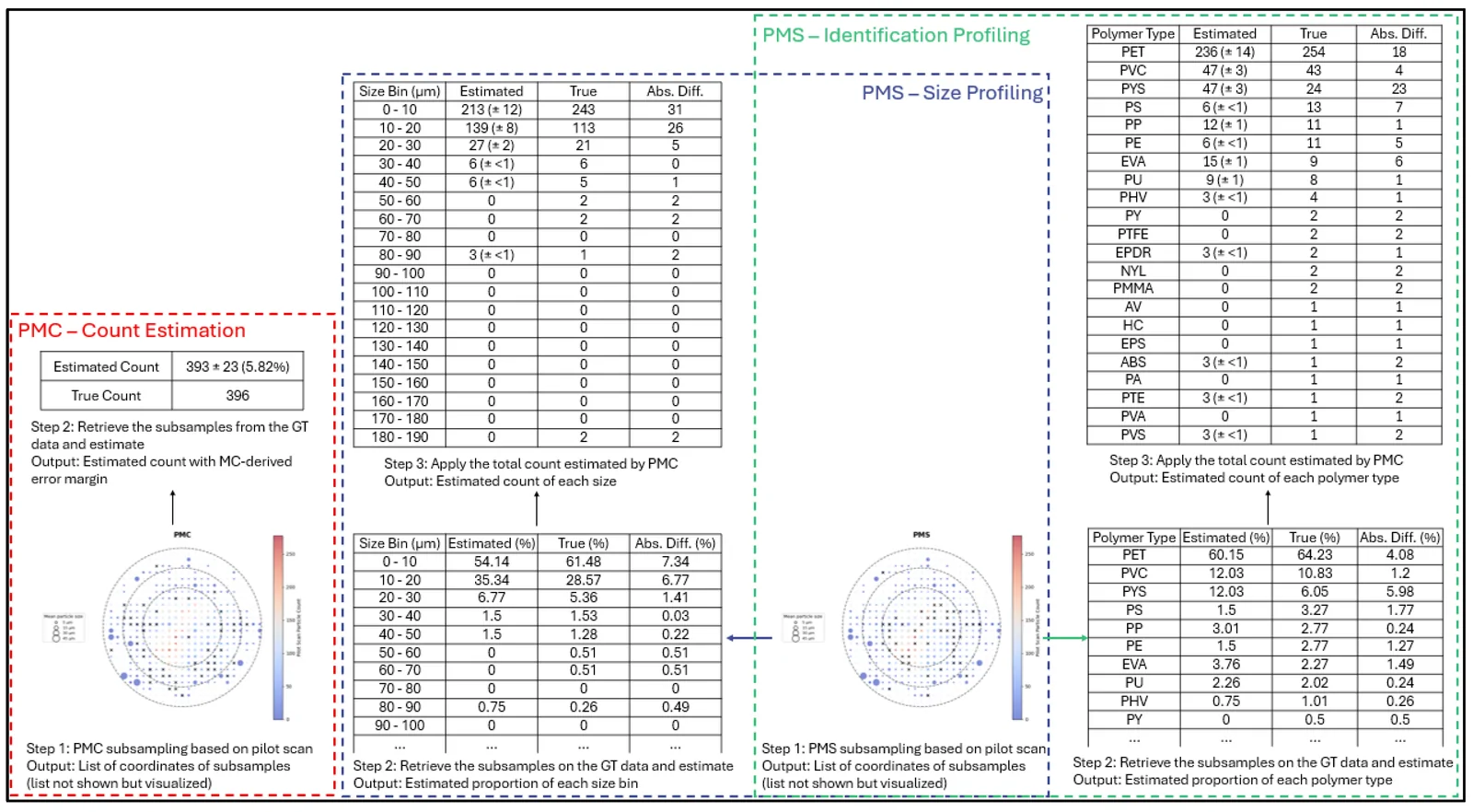
The workflow of the demonstration, including (1) the 2–step process of count estimation by PMC (within red confines), (2) the 3–step process of size profiling by PMS (within blue confines), and (3) the 3–step process of identification profiling by PMS (within green confines).

**Table 1:** Summary of the underlying methodological principles for the subsampling methods across subsample selection, count estimation, and size and polymer type profiling.

Method	Subsampling	Count Estimation	Size Profiling	Identification Profiling
Random	Selects with equal probability.	Means scaled by total locations.	Mean of sampled locations.	Mean of sampled locations.
Stratified	Selects within each tier with equal probability.	Aggregated scaled means from each tier.	Mean of tier-specific profiles weighted by tier areas.	Mean of tier-specific profiles weighted by tier areas.
PMC	Favors locations with counts closest to the global average in pilot scan.	Means scaled by total locations.	- *	Mean of sampled locations.
PPC	Favors locations with high counts in pilot scan.	Horvitz–Thompson weighted sum inverse by inverse probabilities.	- *	Weighted sampled profiles by inverse probabilities.
PMS	Favors locations with Spearman’s rank of size closest to the global in pilot scan.	- *	Mean of sampled locations.	Mean of sampled locations.
PPS	Favors locations with large total particle sizes in pilot scan.	- *	Weighted sampled profiles by inverse probabilities.	Weighted sampled profiles by inverse probabilities.

*: No participation.

**Table 2:** The comparison of the performance of the standard-determined subsampling methods between the standard polymer filters (*n* = 15) and the biosolid sample filter (*n* = 50).

Preferred Method	PMC	PMS			PMS		
Aspect	Count Estimation	Size Profiling			Identification Profiling		
Metric	MRE of Count	MRE of Span	RSE of $D_{50}$	Spearman’s Coefficient	MAE of Polymer Type Proportions	RSD of Most Common Polymer Type	Spearman’s Coefficient
Standard #1	20% ± 4.62%	12%	9%	0.925	6%	16%	0.850
Standard #2	30% ± 9.71%	16%	4%	0.905	9%	35%	0.800
Standard #3	15% ± 3.12%	9%	8%	0.905	6%	19%	0.820
Mean ^1^	22%	12%	7%	0.912	7%	23%	0.823
Biosolid	20%	12%	3%	0.875	0.7%	6%	0.875

^1^ Arithmetic means of the results among the standard polymer filters.

## Data Availability

The original contributions presented in this study are included in the article. Further inquiries can be directed to the corresponding author.

## Supplementary Materials

The following supporting information can be downloaded at https://www.mdpi.com/article/10.3390/toxics14100843/s1, Text S1: Density variability and spatial correlation [43], Text S2: Three criteria in the MC tournament for size profiling, Text S3: Three criteria in the MC tournament for identification profiling, Table S1: Polymer standards used to prepare working stocks, Table S2: Mathematical framework and estimators, and Table S3: Characteristics, i.e., count variability and spatial correlation, of particle distribution, and statistics related to mean local false positive ratios of the filters in the study.
