# Supplementary material for: Evaluation of Subsampling Methods for Micro-Spectroscopy Analysis of Microplastics
Source: Toxics. 2026 Sep 22;14(10):843. doi: 10.3390/toxics14100843 (PMC13709794; doi:10.3390/toxics14100843)
Supplement: Supplementary file 1 [file toxics-14-00843-s001.zip › toxics-4539026-supplementary.pdf]

Supporting Information

Evaluation of subsampling methods for micro-spectroscopy analysis of microplastics

Yifei Wang, Ujjwal Ghimire, Danny Reible and Balaji Rao *

Department of Civil, Environmental, and Construction Engineering, Texas Tech University, Lubbock, TX 79409, USA

* Correspondence: balaji.rao@ttu.edu

Text S1. Density variability and spatial correlation

The heterogeneity of particle distribution was characterized by two properties, namely density variability and spatial correlation.

Density variability was quantified by the Coefficient of Variation (CV):

$$CV = \left(\frac{\sigma_\rho}{\mu_\rho}\right) \times 100\%,$$

where σ_ρ and μ_ρ are the respective standard deviation and mean of local density, ρ_i, which is defined as:

$$\rho_i = \frac{n_i}{a},$$

where n_i is the particle count in the quadrat i, and a is the area of the quadrat i. Lower CV_ρ reflects more even particle distribution.

Spatial autocorrelation measures the degree to which similar values cluster together. It was quantified by Moran's I, which was defined as [43]:

$$I = \frac{n}{W} \frac{\sum_{i=1}^{n} \sum_{j=1}^{n} w_{ij}(x_i - \bar{x})(x_j - \bar{x})}{\sum_{i=1}^{n}(x_i - \bar{x})^2},$$

where n is the total number of quadrats, x_i and x_j are the particle counts in quadrats i and j, $\bar{x}$ is the mean particle count across all quadrats, w_{ij} is the spatial weight between quadrats i and j : w_{ij} = 1 if neighbors (i.e., touching); 0 if not W is the sum of all spatial weights $\sum_i \sum_j w_{ij}$. Moran's I ranges from -1 (i.e., dissimilar values clustered together in a checkerboard pattern) to 0 (i.e., no discernible relationship) to 1 (i.e., similar values clustered together in one hot spot).

Text S2. Three criteria in the MC tournament for size profiling

1. Mean Relative Error of Span (MRE_Span) – Accuracy

$$MRE\_Span = \frac{1}{M} \sum_{i=1}^{M} \left| \frac{Span_s - Span_g}{Span_g} \right| \times 100\%,$$

where M is the number of Monte Carlo iterations, i.e., 1000, and $Span_s$ and $Span_g$ are the spans of the subsample and the ground truth, respectively.

$$Span = \frac{D_{90} - D_{10}}{D_{50}},$$

where D_x is the particle size below which x% of total particles are smaller.

2. Relative Standard Error of D_{50} (RSE_D_{50}) – Precision

$$RSE\_D_{50} = (\frac{\sigma_{D50}}{\mu_{D50}}) \times 100\%,$$

where σ_{D50} and μ_{D50} are the standard deviation and mean of the D_{50} values recorded across all iterations, respectively.

3. Spearman's Coefficient (ρ) – Size Rank Fidelity

$$\rho = 1 - \frac{6\sum d_i^2}{n(n^2-1)},$$

where n is the number of subsampled locations, and d_i is the rank difference in size bin i between the subsample and the ground truth, defined as

$$d_i = rank(s_i) - rank(g_i)$$

Text S3. Three criteria in the MC tournament for identification profiling

1. Mean Absolute Error of Polymer Type Proportions ($MAE\_ID\ Proportion$) – Accuracy

$$MAE\_ID\ Proportion = \frac{1}{n}\sum_{i=1}^{n}\left|P_s - P_g\right| \times 100\%,$$

where n is the total number of unique polymer types in the ground truth, and P_s and P_g are the proportions of a specific polymer in the subsample and the ground truth, respectively.

2. Relative Standard Deviation of Most Common Polymer Type ($RSD\_Key\ ID$) – Precision

$$RSD\_Key\ ID = (\frac{\sigma_{Key}}{\mu_{Key}}) \times 100\%,$$

where σ_{Key} is the standard deviation of the proportion of the most common polymer type across all iterations, and μ_{Key} is the mean proportion of the most common polymer type across those same iterations.

3. Spearman's Coefficient (ρ) – Polymer Type Rank Fidelity

$$\rho = 1 - \frac{6\sum d_i^2}{n(n^2-1)},$$

where n is the number of subsampled locations, and d_i is the rank difference in polymer i between the subsample and the ground truth, defined as

$$d_i = rank(s_i) - rank(g_i)$$

Table S1. Polymer standards used to prepare working stocks.

**Polymer**	**Size Range (μm)**	**Morphology**	**Color**	**Source**
PE	27–32	Sphere	Red	Cospheric LLC (Goleta, CA)

PMMA	1.5–11.5	Sphere	Clear	
PS	9.5–11.5	Sphere	Clear	
PVC	20–30	Fragment	Gray	Dr. Jeffrey M. Farner (FAMU-FSU)

Table S2. Mathematical framework and estimators.

**Method**	**Subsampling**	**Count Estimation**	**Size Profiling**	**Identification Profiling**
Random	$\pi_i = \frac{1}{N}$	$\frac{N}{n}\sum_{i=1}^{n} y_i$	$\frac{N}{n}\sum_{i=1}^{n} \mathrm{P}_i$	$\frac{N}{n}\sum_{i=1}^{n} \mathrm{P}_i$
Stratified	$\pi_{hi} = \frac{1}{N_h}$	$\sum_{h=1}^{L} \frac{N_h}{n_h} \sum_{i=1}^{n_h} y_{hi}$	$\sum_{h=1}^{L} (\frac{N_h}{N} \cdot \bar{\mathrm{P}}_h)$	$\sum_{h=1}^{L} (\frac{N_h}{N} \cdot \bar{\mathrm{P}}_h)$
PMC	$i \in \{j: \lvert w_j - \bar{w} \rvert \leq \delta\}$*	$\frac{N}{n}\sum_{i=1}^{n} y_i$	-	$\frac{N}{n}\sum_{i=1}^{n} \mathrm{P}_i$
PPC	$\pi_i = \frac{w_i + \varepsilon}{\sum w_i}$	$\sum_{i=1}^{n} \frac{y_i}{\pi_i}$	-	$\frac{\sum_{i=1}^{n} \mathrm{P}_i/\pi_i}{\sum_{i=1}^{n} 1/\pi_i}$
PMS	$i \in \{j: \rho(S_j, S_{all}) \geq \tau\}$*	-	$\frac{N}{n}\sum_{i=1}^{n} \mathrm{P}_i$	$\frac{N}{n}\sum_{i=1}^{n} \mathrm{P}_i$
PPS	$\pi_i = \frac{s_i + \varepsilon}{\sum s_i}$	-	$\frac{\sum_{i=1}^{n} \mathrm{P}_i/\pi_i}{\sum_{i=1}^{n} 1/\pi_i}$	$\frac{\sum_{i=1}^{n} \mathrm{P}_i/\pi_i}{\sum_{i=1}^{n} 1/\pi_i}$

* Subsampling from the eligible pool of locations.

Parameter Glossary for Table S1

π_i: Probability of location i to be subsampled.

N, n: Total number of locations and number of sampled locations, respectively.

y_i: The ground truth count in location i.

P_i: The vector representing the local profile (size distribution or polymer identification composition) in location i.

w_i, $\bar{w}$: The local pilot count in location i and global pilot mean count, respectively.

s_i: Local pilot particle length sum based on sizes.

N_h, n_h: Total number of total locations in stratum h, and number of sampled locations in stratum h, respectively.

ε: Numerical constant (10^{-2}) to ensure non-zero inclusion probability.

δ: Threshold of absolute count deviation of the local from the global mean; 25th percentile applied.

τ: Threshold of Spearman's coefficient between local and global size histograms; 75th percentile applied.

S_j, S_{all}: Local and global size histograms, respectively.

Table S3. Characteristics, i.e., count variability and spatial correlation, of particle distribution, and statistics related to mean local false positive ratios of the filters in the study.

**Filter**	**Count Variability (CV)**	**Spatial Correlation (Moran's I)**	**Mean Local False Positive Ratio***	**Relative Standard Error of Mean Local False Positive Ratio**
Standard Polymer #1	109.31%	0.3086	54%	66.19%
Standard Polymer #2	164.31%	0.5275	60%	65.19%
Standard Polymer #3	96.63%	0.3807	74%	33.98%
Biosolid Sample	222.88%	0.5854	98%	5.08%

* Local false positive ratio (FPR) is calculated as

$$Local\ FPR_i = \frac{w_i - y_i}{w_i},$$

where w_i and y_i are the respective counts in the pilot scan and the ground truth in location i.
